# The Effect of Nano-ZnO on Seeds Germination Parameters of Different Tomatoes (*Solanum lycopersicum* L.) Cultivars

**DOI:** 10.3390/molecules27154963

**Published:** 2022-08-04

**Authors:** Katarzyna Włodarczyk, Beata Smolińska

**Affiliations:** Institute of Natural Products and Cosmetics, Department of Biotechnology and Food Sciences, Lodz University of Technology, Stefanowskiego Str. 2/22, 90-537 Lodz, Poland

**Keywords:** tomato, NPs, nano-ZnO, seeds germination

## Abstract

The agriculture sector faces numerous problems. One of the beforementioned problems relates to the proper crop plants’ fertilization. The conventional bulk fertilizers are becoming less effective and have a negative impact on the environment. Nanomaterials such as zinc oxide nanoparticles (ZnO NPs) are widely used in various sectors such as medicine or electronics. Several studies indicate that nano-ZnO may likewise be considered as a potential nanofertilizer. In present research, an attempt was made to study the influence of two different sized ZnO NPs (<50 nm and <100 nm) on the seed germination of chosen tomato (*Solanum lycopersicum*) cultivars. The seeds of three cherry tomato cultivars were placed on a Petri dish with the NPs suspensions (0, 50, 150, and 250 mg/L) in order to examine the influence on germination parameters at a certain size of NPs and at a chosen concentration. In addition, within this study, we verified that the implicated conditions have the exact impact on all three cultivars. The obtained results indicate that all the factors affect the seed sprouting, however, this process mainly depends on the type of tomato cultivar and the size of the used nanoparticles. The parameter of the germination percentage (GP) was the only of the assumed factors that did not influence it significantly. Nevertheless, the values of other examined parameters such as the MGT, GRI, CVG, or VI depend strongly on all assumed features including the type of chosen cultivar. The obtained results vary significantly between all cultivars which indicates that the plants from the same family may require different conditions for optimal growth. In this research the <50 nm ZnO nanoparticles had more beneficial influence on sprouting parameters then parallelly used <100 nm ZnO nanoparticles.

## 1. Introduction

The fast development of the world population in the past few decades has pushed the agricultural sector to increase crop productivity to satisfy the needs of billions of people. The agricultural zones are reaching the limits of their natural material availability, which results in a stagnation in crop yield, declining soil organic matter, and water availability [1]. The Food Agriculture Organization of the United Nations (FAO) collected the data that indicate the exhaustion and degradation of land and water. This situation creates major challenges such as the production of food for the world’s increasing population. It is projected that in 2064, the human population will increase to up to 9.73 billion people [2]. This situation will force the industry to double food production to fulfill the consumers’ needs. While soil is the most favorable for crop growth, open-field agriculture might face significant problems such as the limited availability of land, agriculture productivity, or extensive environmental pollution.

The most severe problem for proper agriculture is the low efficiency and the environment contamination caused by the wide use of conventional bulk fertilizers. Basic fertilizers are nitrogen- and phosphorus-based and both of these compounds can easily change their chemical forms to the ones which are not accepted by plants. Consequently, a large number of fertilizers (50–70%) [3,4] is lost to the atmosphere or surface water bodies, causing pollution. They can additionally be washed by rain into rivers and lakes, where they cause contamination.

Essentially, it is necessary to create a safer way of manuring the soil, for instance, using nanofertilizers. During the last two decades, nanotechnology has revolutionized numerous industrial and academical fields such as medicine, material science, electronics, and engineering. Nanoparticles (NPs) (1 to 100 nm) have specific physical, chemical, and mechanical properties in comparison with their bulk form [4]. Many studies have been carried out to apply nanotechnology, among others, in the form of nanofertilizer and nanopesticide in the agriculture sector. NPs such as Ag, Ti, Si, Zn, Cu, Fe, and their oxide or carbonanotubes are constantly examined as potential fertilizers or as a support for conventional fertilizers [5,6,7,8,9,10,11]. However, the data on the subject are not complete and there are still plenty of contradictions in terms of the beneficial roles of NPs in the agriculture sector. In some research, the results indicate that NPs are able to stimulate plants’ growth, whereas in other studies, it is presented that they contribute to decreased root length [10,12]. It was further proven that a variety of plants can accumulate NPs in their tissue [13,14,15]. One of the advantages of using NPs in plants fertilization is a possibility to obtain the controlled release of chosen macro- or micronutrients to the soil, which could be effective in reducing the environmental pollution [16]. 

One of the frequently used metal oxide NPs in the industry is nano-ZnO (zinc oxide nanoparticles). Thus, for the last two decades the research on how this nanoparticle affects the plants has been still ongoing. Several studies have provided the proof for the beneficial effects of ZnO NPs on chosen plants. For instance, in 2007, Lin and Xing [17] reported the advantageous influence of those NPs on the growth of radishes (*Raphanus sativus*) and rapeseed (*Brassica napus*). The application of low concentrated ZnO NPs (2 mg/L) enhanced the germination of seeds of the abovementioned plants and boosted root elongation as well. Later, Mahajan et al. (2011) [18] conducted the research where they examined the influence of ZnO NPs on the growth of mung (*Vigna radiate*) and chickpea seedlings (*Cicer arietinum*). In this research, ZnO NPs revealed positive effects by increasing the length and the biomass of the roots and shoots of both plants. Another example of positive effects of ZnO NPs was reported by Zhao et al. (2014) [18]. ZnO NPs aided to obtain a higher root and fruits dry mass (respectively, 1.1–1.6 and for fruits, 0.6 higher compared to the control) examined in cucumber (*Cucumis sativus*). However, for different plants, different concentrations of ZnO NPs are toxic. This is due to the fact that NPs can accumulate in plant tissues which can affect the physiological and biochemical processes.

A further popular plant on which the influence of ZnO NPs was examined is the tomato (*Solanum lycopersicum*). In 2013, de la Rosa et al. [19] published a paper on the influence of different concentrations of a suspension of nano-ZnO (0, 50, 100, 200, 400, 800, and 1600 mg/L). In the presented research, they proved that the suspension at a concentration of 1600 mg/L of ZnO NPs reduced the germination percentage of tomato seeds by 20%. Moreover, the root growth of seedlings likewise decreased (by 50%) at a ZnO NPs concentration of 800 and 1600 mg/L. Even so, with the application of 800 mg/L ZnO NPs, seedlings increased the biomass production by 35% with respect to the controls. 

Additionally, in the research of Khanm et al. (2018) [20], it was explained that the treatment with lower concentrations of nano-ZnO may lead to better results. For instance, seeds treated with the application of 400 ppm ZnO NPs obtained a high seed germination percentage (93.33%), as well as the Seedling Vigor Index (919.80), compared to the control. Treatment with concentrations higher than 400 ppm caused a decrease in the Seedling Vigor Index.

Amooaghaie et al. (2017) [21] investigated the influence of both Zn and ZnO NPs on tomato and wheat germination and growth. Results of this study revealed that Zn and ZnO NPs at lower concentrations (for ZnO NPs it is 50 mg/L) promote seed germination and boost the growth parameters, while higher concentrations lead to a decrease in the examined parameters. Faizan et al. (2019) [22] conducted a similar research on the examination of the effects of foliar spraying on tomatoes. The concentrations of nano-ZnO used in this research were 10, 50, 100, and 200 ppm. The suspension of ZnO NPs at 50 ppm were the most influential. The following parameters increased: shoot length by 30.1%, shoot fresh mass by 27.7%, shoot dry mass by 29.0%, root length by 28.7%, root fresh mass by 26.1%, and root dry mass by 24.6%. Additionally, parameters such as leaf area, chlorophyll content, the fruit number per plant, and fruit yield obtained better results comparing to other higher concentrations of the ZnO NPs suspension.

Nonetheless, even now there is some inaccuracy between the provided results of the mentioned studies. Firstly, the concentrations of ZnO NPs used in these different studies vary significantly from 10 ppm (very low) to 1600 mg/L. Secondly, the researchers are using NPs of various sizes, which may be one of the reasons why the delivered results differ considerably. Even though many studies revealed the promising effects of nanofertilization, the data are not yet complete and the mechanisms of the possible phytotoxicity of NPs on certain plants remain unknown.

To provide more data on the potential influence of nano-ZnO on tomatoes (*Solanum lycopersicum*), we decided to examine the influence of different sized NPs (<100 nm and <50 nm) on three different cherry tomato cultivars, Maskotka (W.Legutko, Jutrosin, Poland), Granit, and Malinowy Bossman (PlantiCo, Warsaw, Poland). Additionally, tomato seeds were treated with ZnO NPs suspensions of different concentrations (50 mg/L, 150 mg/L, 250 mg/L).

This study aimed to evaluate the effect of different sizes and concentrations of NPs on the seed germination of the selected tomato cultivars. To the best knowledge of the authors, this is the first study investigating and focusing on the influence of chosen ZnO nanoparticles on the germination parameters of different cultivars of the same plant.

## 2. Results

**Figure 1:**
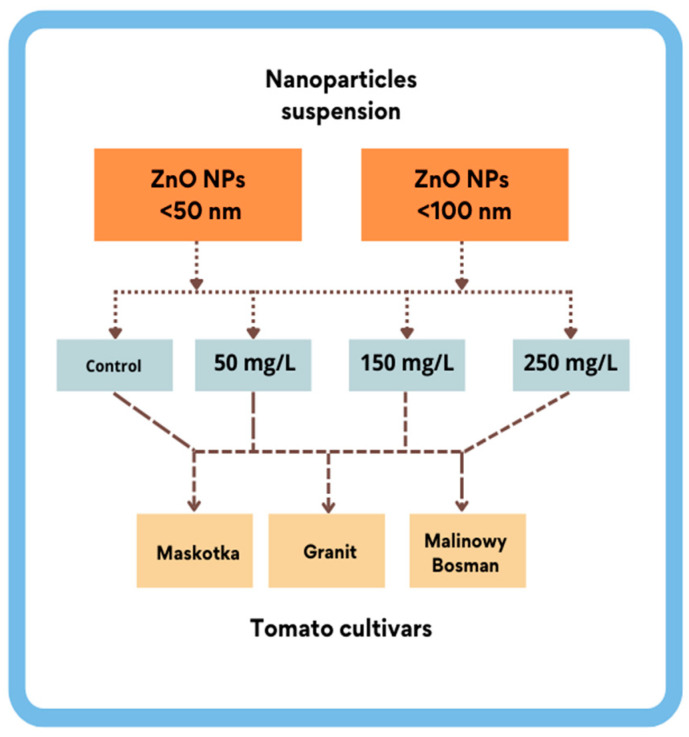
Illustration of research scheme.

In this research, the influence of nano-ZnO on tomatoes was measured on three different tomato cultivars with NPs suspensions at three different concentrations. Each of the chosen cultivars belong to cherry-tomato-type tomatoes. Moreover, two different-sized NPs of nano-ZnO (≤100 nm and ≤50 nm) were used in order to disclose if this parameter may additionally influence the parameters related with seed germination (Figure 1).

### 2.1. Germination Percentage (GP)

**Figure 2:**
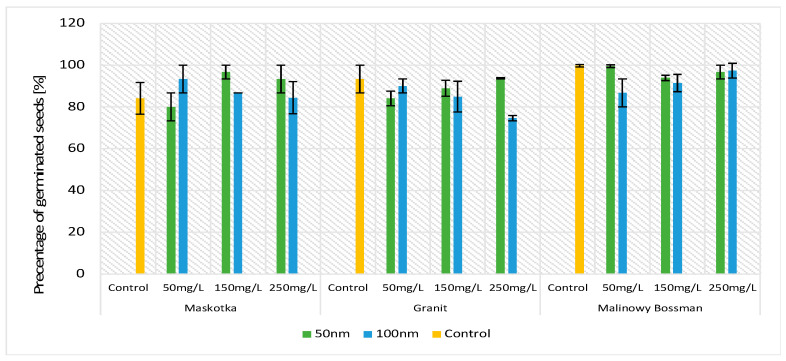
Effect of zinc oxide NPs on the germination percentage (GP) of tomato seeds after 5 days of incubation. All data are the mean of 3 replicates and are demonstrated on vertical bars (±SD). It was calculated that there are no significant differences between the treatments at *p*-value ≤ 0.05.

The germination percentage is a crucial parameter in terms of tomato seeds sprouting. The obtained results indicate that of all three assumed factors (cultivars, concentrations, and NPs’ size), only one had an influence on the GP parameters compared with the controls (Figure 2). The obtained data indicate that for all implemented factors, the results vary mainly between the types of cultivars. Comparing the values of the GP based on the concentration of the used NPs solution or on the particle size factor, the GP values do not vary significantly (except for the Granit cultivar at a concentration of 250 mg/L). This observation may lead to the conclusion that ZnO NPs do not disrupt the process of sprouting. Statistical analysis confirms that observation (Figure 2).

During the experiment, approximately 10 to 15 seeds germinated on each plate. For the cultivar Granit treated with 250 mg/L ZnO NPs (<100 nm), the mean result of germination was 10 seeds per plate, and for the cultivar Malinowy Bossman, it was 15 germinated seeds at a concentration of 50 mg/L (<50 nm) per plate. The highest GP was received for the Malinowy Bosman cultivar treated with <50-nm-size NPs (GP equal over 90% for all used concentrations), whereas the Granit cultivar presented the lowest growth while being treated with <100 nm size NPs (74.6% GP at a concentration of 250 mg/L).

### 2.2. Mean Germination Time (MGT)

**Figure 3:**
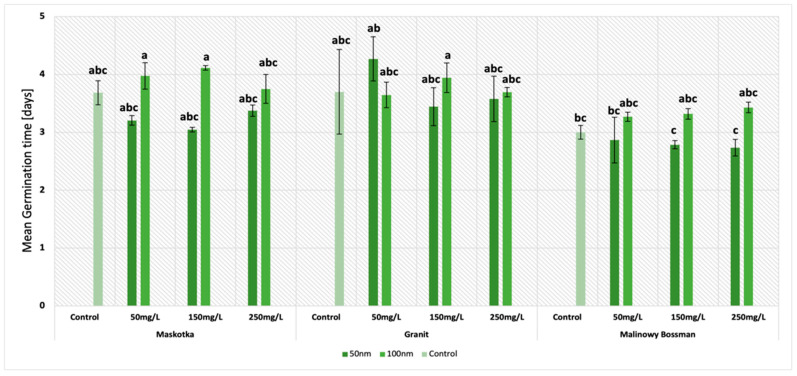
Effect of zinc oxide NPs on the mean germination time (MGT) of tomato seeds after 5 days of incubation. All data are the mean of 3 replicates and vertical bars demonstrate standard deviation (±SD). Different letters indicate the significant differences for *p*-value ≤ 0.05 (Table A1).

The second important parameter which was examined in this research was the mean germination time (MGT). The results obtained in this study indicate that two factors had a significant influence on the MGT of tomato seeds. The first was the particle size and the second was the type of cultivar. It is worth noting that regardless of the cultivars, (except Granit at conc. 50 mg/L) the NPs of the size < 50 nm provided lower results of the MGT (Figure 3). This tendency is well illustrated by the results obtained for the cultivar Maskotka. At a concentration of 50 mg/L (<50 nm NPs), the value of the MGT was 20% lower than the one exhibited by the seeds of the same cultivar treated with <100 nm NPs. The same trend was observed with the use of higher concentrations of NPs. For suspensions at concentrations of 150 mg/L and 250 mg/L, the results were lower by 19% and 11%, respectively. Similarly, the MGT results obtained by the usage of a suspension of <50 nm NPs are likewise lower when compared to the control. Interestingly, the MGT values obtained by the Maskotka cultivar treated with <100 nm NPs are higher than the results of the control, which indicates the negative impact of the beforementioned NPs on the MGT parameter for tomato seeds. The results for the two other cultivars indicated the same tendency.

The other mentioned feature that has affected the MGT results was the type of cultivar. The disparity between cultivars is considerable. For example, at a concentration of 250 mg/L (NPs < 50 nm), the lowest result was obtained by the cultivar Malinowy Bossman, while the MGT of the Maskotka and Granit cultivar at the same conditions were 26% and 33% higher, respectively. The trend is similar for other concentrations, even for the suspension of <100 nm NPs. At a concentration of 150 mg/L (NPs < 100 nm), the lowest MGT was obtained again by Malinowy Bossman, and the results of the Maskotka and Granit cultivars were 24% and 18% higher, respectively. Nevertheless, the tendency has demonstrated that lower results were obtained with the treatment of <50 nm NPs. The Maskotka cultivar was the one in which the treatment of NPs (<50 nm) significantly reduced the mean germination time of the seeds. In comparison to the control, the MGT of Maskotka was reduced by 13.5% at a concentration of 50 mg/L, 19% at a concentration of 150 mg/L, and 8% at a concentration of 250 mg/L. Interestingly, the concentration of the used NPs suspensions did not have an essential impact on the results of the MGT, except for the Granit cultivar which reached the highest value of MGT of all the cultivars at a concentration of 50 mg/L.

### 2.3. Germination Rate Index (GRI)

**Figure 4:**
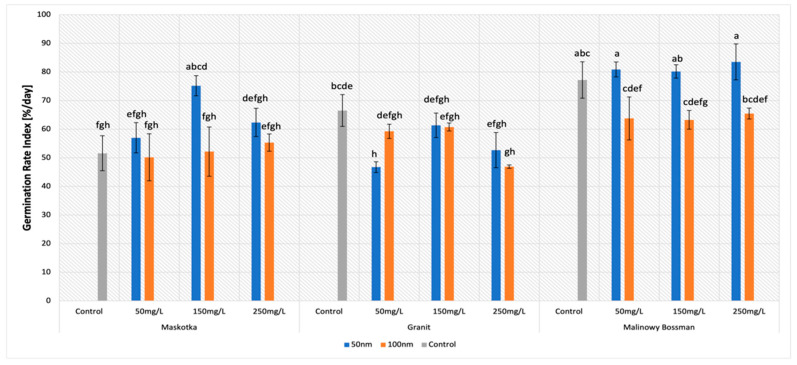
Effect of zinc oxide NPs on the germination rate index (GRI) of tomato seeds after 5 days of incubation. All data are the mean of 3 replicates and vertical bars demonstrate standard deviation (±SD). Different letters indicate the significant differences for *p*-value ≤ 0.05 (Table A2).

The germination rate index is the parameter which reflects the percentage of germination of seeds during each day. In this research, all assumed features had a strong impact on the values of GRI parameters (Figure 4). The statistical analysis indicated the importance of all applied factors. The major impact on the GRI was the type of cultivar (*p*-value < 0.001) and the NPs size (*p*-value < 0.001), however, this time, the concentration of the NPs suspensions also affected the GRI (*p*-value ~ 0.001–0.01).

**Figure 5:**
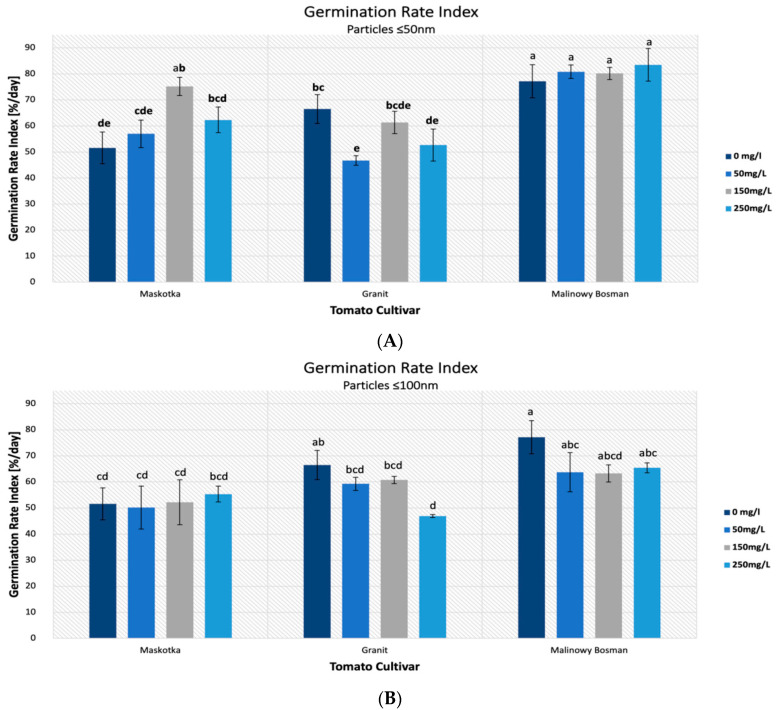
Effect of zinc oxide NPs (<50 nm) (**A**) and (<100 nm) (**B**) on the germination rate index (GRI) of tomato seeds after 5 days of incubation. All data are the mean of 3 replicates and vertical bars demonstrate standard deviation (±SD). Different letters indicate the significant differences for *p*-value ≤ 0.05 (Table A3).

The highest results of the GRI were mostly achieved with the use of the suspensions of <50 nm NPs. For the cultivars Maskotka and Malinowy Bossman, results obtained from treatment with the use of <50 nm NPs was significantly higher than with the usage of <100 nm ZnO NPs (Figure 5A) and noticeably higher than the controls. For the Maskotka cultivar, the suspension of NPs (<50 nm NPs) at a concentration of 150 mg/L was optimal, and the obtained value of the GRI was 45.7% higher than the control and 44% higher than the suspension at the same concentration, but with <100 nm NPs. Interestingly, the treatment of <100 nm NPs, in most cases, led to a lower GRI value compared with the control (Figure 5B), which suggests that the <100 nm NPs have a negative impact on tomato seeds. In general, for the Maskotka cultivar the usage of <50 nm NPs resulted in an increase in the GRI. In contrast, for the same cultivar, the treatment with <100 nm had an adverse effect. For the Malinowy Bossman cultivar, the situation is similar. However, the result of <50 nm NPs treatment is not notably higher than the GRI of the control. For the Granit cultivar, each treatment had an adverse effect on the seeds, regardless of the concentration of the used suspension or the size of the NPs. The results after treatment were considerably lower than from the control. Surprisingly, the GRI value obtained after the treatment of the <50 nm NPs suspension, even at the concentration of 50 mg/L, were lower by 30% from the control. This is the only case in which the <50 nm NPs suspension caused a decrease in the GRI.

### 2.4. Coefficient of Velocity of Germination (CVG)

**Figure 6:**
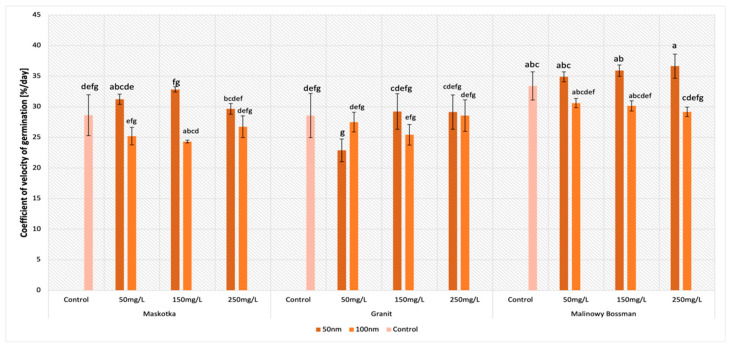
Effect of zinc oxide NPs on the coefficient of velocity of germination of tomato seeds after 5 days of incubation. All data are the mean of 3 replicates and vertical bars demonstrate standard deviation (±SD). Different letters indicate the significant differences for *p*-value ≤ 0.05 (Table A4).

The parameter of the coefficient of velocity of germination (CVG) in general gives an indication of the rapidity of germination. The received data indicate that the concentration of the used ZnO NPs suspensions had no significant influence on the CVG in this study (Figure 6). Nevertheless, factors such as the type of cultivar or the size of the NPs affect this parameter significantly. The highest values of the CVG were achieved by the Maskotka cultivar when the seeds were treated by <50 nm ZnO NPs. Seeds incubated with the NPs suspensions (<50 nm) at the concentration of 150 mg/L reached a 14.7% higher result than the control. Additionally, the ZnO NPs suspension at concentrations of 50 mg/L and 250 mg/L led to obtaining higher results than the control by 9.1% and 3.5%, respectively.

For the Granit cultivar, the values of the CVG vary significantly, only the <50 nm suspension at concentrations of 150 mg/L and 250 mg/L issued slightly higher result than the control. Nevertheless, differences between the abovementioned results and the controls reached approximately 2%. For other treatments, the results are lower than the control or at the same level (for the <100 nm suspension at a conc. of 250 mg/L). In the case of the Malinowy Bossman cultivar, the ZnO NPs < 100 nm reduced the CVG. Regardless of the used concentration, the values of the seeds treated with the ZnO NPs suspension were lower than the control. In contrast, smaller ZnO NPs (<50 nm) revealed an adverse effect and the results from all used suspensions at different concentrations were greater than the value of the control. The results for the 50, 150, and 250 mg/L suspensions reached higher values than the control by 4.5%, 7.5%, and 9.6%, respectively.

### 2.5. Dry Weight (DW)

**Figure 7:**
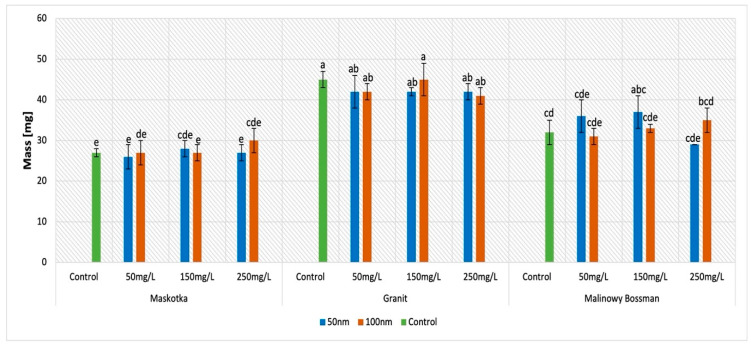
Effect of zinc oxide NPs on the dry weight of germination of tomato seeds after 5 days of incubation. All data are the mean of 3 replicates and vertical bars demonstrate standard deviation (±SD). Different letters indicate the significant differences for *p*-value ≤ 0.05 (Table A5).

The results obtained for the examination of dry weight indicates that mainly the cultivar type influences this parameter (Figure 7). Interestingly, the mass of the seeds which were under the treatment of the ZnO NPs suspensions, independently from the concentration of the suspension or the size of the NPs, is similar to the mass of the corresponding controls. Although for the Maskotka cultivar, differences between the mass of the samples and the control are inconsiderable, for the other two cultivars, the situation is slightly different. For instance, for the Granit cultivar, all results are lower than the control (except for ZnO NPs < 100 nm at a conc. of 150 mg/L) by approximately 7%. In contrast, the suspension of <50 nm NPs at concentrations of 50 and 150 mg/L provided higher results by 12.5% and 15.6%. Interestingly, for this cultivar, the results provided by the treatment of <100 nm NPs are higher than the control as well, except for the seeds treated with ZnO NPs at a concentration of 50 mg/L. The obtained results are higher than the control by 3% for the 150 mg/L concentration of the used suspension and by 9.4% by the 250 mg/L suspension.

### 2.6. Vigor Index (VI)

**Figure 8:**
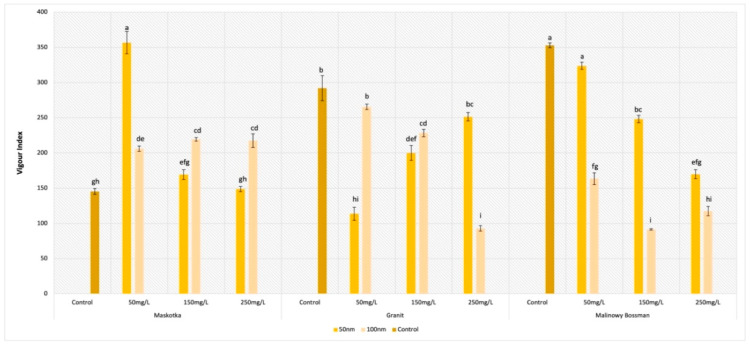
Effect of zinc oxide NPs on the vigor index germination of tomato seeds after 5 days of incubation. All data are the mean of 3 replicates and vertical bars demonstrate standard deviation (±SD). Different letters indicate the significant differences for *p*-value ≤ 0.05 (Table A6).

**Figure 9:**
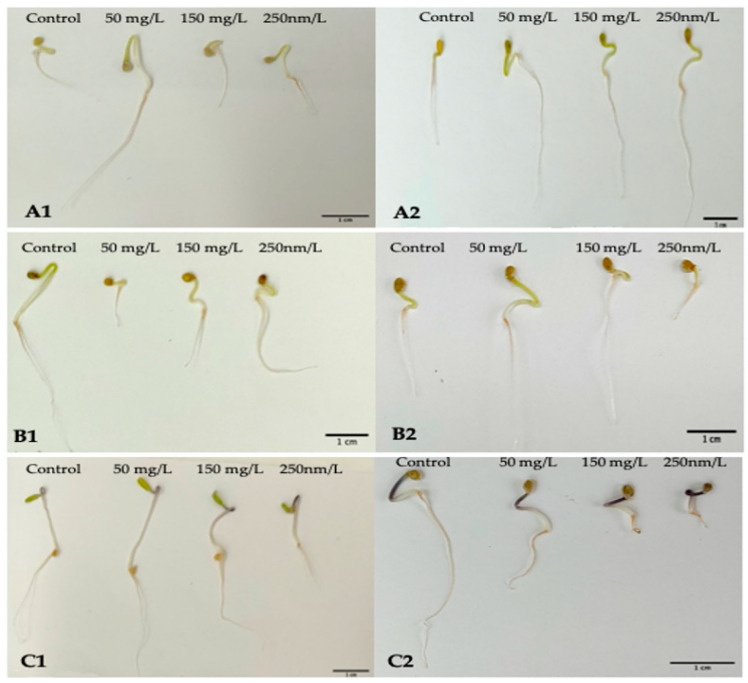
The picture of seedlings after 5 days of incubation. The seedlings of Maskotka cultivar treated with the suspensions of NPs (**A1**) <50 nm and (**A2**) <100 nm. The seedlings of Granit cultivar treated with the suspensions of NPs (**B1**) <50 nm and (**B2**) <100 nm. The seedlings of Malinowy Bossman cultivar treated with the suspensions of NPs (**C1**) <50 nm and (**C2**) <100 nm.

For the parameter of the vigor index (VI), all assumed factors had a significant influence on the obtained results (Figure 8). For the Maskotka cultivar, the ZnO NPs suspensions, regardless of the concentration or the size of the NPs, had a positive impact on the VI. The highest value of VI was reached by the seeds treated with NPs < 50 nm at a concentration of 50 mg/L. Obtained results for those seeds were 145.5% higher than for the control (Figure 9(A1,A2)).

Unexpectedly, for the seeds of Maskotka treated with higher concentrations of the ZnO NPs suspension, the results showed other tendencies. At concentrations of 150 and 250 mg/L, the higher results were obtained by seeds treated with the <100 nm NPs. Nevertheless, all obtained results for the Maskotka cultivar were increased compared to the control. This observation could lead to the conclusion that ZnO NPs treatment induces the VI parameter for the Maskotka cultivar. Interestingly, for two other cultivars, the situation is slightly different. For the Granit cultivar, all used treatments diminish the VI (Figure 9(B1,B2)). The lowest results were obtained by the usage of <50 nm NPs at a concentration of 50 mg/L and <100 nm NPs at a concentration of 250 mg/L, which were lower than the control by 60% and 78%, respectively. None of the used concentrations of ZnO NPs, independently of the NPs’ size, affected the Granit seeds positively in terms of the VI parameter. A similar phenomenon was observed with the Malinowy Bossman seeds. None of the used suspension resulted in the increase of the VI value or in higher results than the control. The difference between the VI value of the control and the VI of the experimental group of the 50 mg/L (<50 nm) NPs suspension was minor (8%). An interesting tendency was observed for the <50 nm suspensions, as the obtained results of VI were decreasing steadily with the increase in the concentration (Figure 9(C1,C2)).

## 3. Discussion

In our research, we proved that while using the ZnO NPs solution to improve seeds sprouting, it is crucial to pay attention to the selection of factors such as the size of the chosen NPs, the concentration of the used solution, and the choice of cultivar type. The data obtained in this research allow for certain conclusions.

The results of the GP suggest that the beforementioned parameters vary for different types of examinate cultivars, but the other implemented factors such as the size of the used NPs or the concentration of the ZnO NPs suspension do not significantly influence the final result. This observation is similar to the study of de la Rosa (2013) [19] and Raliya et al. (2015) [23]. In the first study, the size of the nano-ZnO was approximately 8 nm, but aside from the particle size, in the abovementioned research, there were five different concentrations of ZnO NPs suspensions. Nevertheless, none of the used concentrations, except 1600 mg/L, affected seed germination. In the research of Raliya et al. (2015) [23], the used NPs were 25 nm and the concentration of the NPs suspensions were likewise lower than in the first mentioned article. The outcome of this study proved that nano-ZnO NPs are toxic for tomato seeds when the concentration of the NPs suspension is equal to or higher than 750 mg/kg. At the same time, in this study, it was demonstrated that ZnO NPs at a concentration lower than 750 mg/kg do not inhibit seed germination. Those conclusions are confirmed in our study. Even so, the differences between the obtained GP values are not significant. The results vary between the cultivars, but the GP values obtained after NPs’ treatment are not considerably different from the controls.

Interestingly, the MGT values were different for all types of cultivars. This phenomenon may be seen for the control as well. Nevertheless, even though there were differences in the results of MGT between the types of used cultivars, still, for all of them, the specific tendency has been kept. As it was mentioned above, for all cultivars (except the Granit cultivar at a concentration of 50 mg/L), the use of smaller ZnO NPs (<50 nm) resulted in a lower MGT and, to the contrary, the use of ZnO NPs at the size < 100 nm caused the obtaining of higher values of MGT than in the control. This may lead to the conclusion that, firstly, the application of ZnO NPs < 50 nm positively affects the seeds, and secondly, the usage of smaller NPs is more beneficial than the usage of the <100 nm ZnO NPs. The reduction of the seeds germination time confirms this observation. In the study of Panwar et al. [24] they proved that <100 nm NPs are not toxic for tomatoes up to the concentration of 250 mg/L. The authors only considered the germination percentage in terms of the seeds’ sprouting parameters. This conclusion is also confirmed in our study. Nevertheless, MGT obtained with the treatment of <100 nm ZnO NPs were similar to the values obtained by the controls. This allows us to conclude that <100 nm ZnO NPs do not influence the mean germination time, while <50 nm ZnO NPs reduce it noticeably. The Maskotka cultivar is the best example of observation, given that the treatment of NPs (<50 nm) significantly reduced the mean germination time of seeds in all used suspensions. The other feature that affects the MGT was the type of used cultivar. This trend was maintained for both <50 nm and <100 nm ZnO NPs. For instance, for the Malinowy Bossman cultivar, the suspension at a concentration of 250 mg/L (NPs <50 nm) led to a decrease in the MGT, while for the two other cultivars, the same conditions caused considerably higher results. Again, the Malinowy Bossman cultivar at a concentration of 150 mg/L (<100 nm) NPs obtained the lowest result, and the results of the Maskotka and Granit cultivars were higher by approximately 20%.

Another parameter that was examined in our research is the GRI. In general, the best results for this parameter were achieved with the use of <50 nm ZnO NPs. For cultivars Maskotka and Malinowy Bossman, values of the GRI obtained for the treatment of the <50 nm ZnO NPs was much higher than for the <100 nm ZnO NPs and noticeably higher than for the controls. Interestingly, the treatment of the <100 nm ZnO NPs in most cases led to a lower GRI value compared to the control. That observation may lead to a conclusion that larger-sized ZnO NPs may be assumed as toxic for tomato seeds. As the germination rate index is the parameter that relates to the percentage of the germination of seeds during each day, this conclusion coincides with the results of the MGT. However, in the case of the GRI, the statistical analysis helps to notice that all applied features influence the results. As it was mentioned above, the usage of <50 nm ZnO NPs caused higher results for the Maskotka and Malinowy Bossman cultivars. At the same time, the use of <100 nm ZnO NPs ensued in obtaining lower GRI values. Interestingly, the seeds of the cultivar Granit reacted adversely for the beforementioned treatment. For this cultivar, <50 nm ZnO NPs as well as <100 nm ZnO NPs caused a decrease in the GRI values. The third factor which affected the results was the used concentrations of the NPs suspension. For the Maskotka cultivar, the concentration of <50 nm ZnO NPs had a significant value. The highest result was obtained with the use of the 150 mg/L suspension, then for the 250 mg/L concentration, and surprisingly, the worst for the 50 mg/L. For the same cultivar, when the <100 nm ZnO NPs were used, the concentration of the suspension did not affect the results significantly. Inversely, in the case of the Malinowy Bossman cultivar, regardless of the used concentration, the tendency was constant. The usage of the <50 nm ZnO NPs gave slightly higher results than the control, but was similar for all used concentrations. The usage of the <100 nm ZnO NPs caused a substantial diminution of the GRI, but the results for all concentrations, except the control, were comparable. For the Granit cultivar, even though the GRI was lower than for the control, the usage of different concentrations of <50 nm brought different results. While for the <100 nm ZnO NPs, the suspension at 50 mg/L and 150 mg/L concentrations provided the same results, and only the 250 mg/L suspension caused a greater decline of the GRI value. The comparison of the results of the GRI parameter clearly indicates that studies on the influence of different types and sizes of NPs on plants must be more extended. In the research of Siddiqui and Al-Whaibi [25], the influence of different concentrations of SiO_2_-NPs on tomato seeds was measured. In this study, authors proved that different concentrations of an NPs suspension affect the germination parameters of tomato seeds. The treatment of 8 g/L nSiO_2_ provided the best results and increased parameters such as nSiO_2_-increased seed germination by 22.16%, germination mean time by 3.98%, the Seedling Vigor Index by 507.82%, and the Seed Germination Index by 22.15%, compared to the controls. Nevertheless, in this study, only one type of cultivar was examined, which causes a problem in comparing the results from that research with our findings. However, it still proves that the concentration of the used suspension may influence the results of sprouting.

The results obtained for CVG are more consistent than previously described parameters. In the case of CVG, mainly the type of cultivar or the size of ZnO NPs affect this parameter significantly. For cultivars Maskotka and Malinowy Bossman, the usage of <50 nm ZnO NPs at most concentrations caused slightly higher results than the control, while the use of <100 nm ZnO NPs provided lower values of CVG than for controls. For the Granit cultivar, the results vary significantly, and no clear tendency is seen. Nevertheless, based on these results, it may be concluded that ZnO NPs affect the CVG of tomato seeds. For instance, in another study, Sobarzo-Bernal et al. [26] proved that the Ce particles do not affect the CVG of tomato seeds. In this study, the seeds were incubated with the Ce particles at different concentration (5, 10, and 15 mg/L). The usage of Ce at a concentration of 5 mg/L increased the CVG compared to the control, although the difference is not significant. In another study, Mohammed et al. [27] used silica nanoparticles on cucumber seeds at four different concentrations (100, 200, 300, and 400 mg/L). The treatment of Si-NPs, regardless of the concentration, enhanced the parameters connected with germination and growth development. The most optimal concentration of the Si-NPs suspension was 200 mg/L. The usage of this suspension caused a 41.4% increase in the CVG for those seeds. Higher concentrations of Si-NPs also increased the CVG and other parameters in comparison to the control. Those studies clearly indicate that the gathered data on the influence of the selected NPs on different plants are still not complete.

Another parameter of seeds germination that was measured in this study was the dry weight of seeds. The only factor that significantly affected the results of this measurement was the type of cultivar. The mass of the seeds which were under the treatment of the NPs suspensions were similar to the mass of the corresponding control. This observation indicates that ZnO NPs may not have an influence on biomass of seedlings. A similar observation was noted by de la Rosa et al. [19], where it was proven that the DW of tomato seedlings was not increased until the seeds were treated with 800 mg/L ZnO NPs. A lower concentration of ZnO NPs increased the biomass, but not significantly. In the research of Wang et al. [28], the ZnO NPs at a concentration 400 mg/L had decreased shoot and root dry masses of tomato by approximately 10%, while the 800 mg/L treatment caused about 50% lower result. Nevertheless, these study measurements were taken on 5-week-old plants. The abovementioned research, as well as this study, may confirm that ZnO NPs at concentrations of up to approximately 800 mg/L do not change the dry mass of tomato seedlings.

The last parameter of seeds germination that was calculated in this research was the Vigor Index. The most significant result was obtained by the cultivar Maskotka which was treated with the <50 nm NPs at a concentration of 50 mg/L. The obtained VI was higher than the control by 145.5%. For the two other cultivars, the treatment of the NPs suspension at 50 mg/L gave the highest results as well, but at the same time, those values were slightly lower than the controls. Additionally, for Granit, the highest result was obtained with the use of <100 nm NPs in contrary to the two other cultivars. For the Malinowy Bossman cultivar, the differences between the results obtained by the <50 nm and <100 nm NPs are considerably high, with more beneficial action at <50 nm. Still, the usage of a higher concentration of ZnO NPs resulted in outcomes lower than the control. Interestingly, the VI of the Granit cultivar varies among the different concentrations of NPs suspensions. At a concentration of 50 mg/L, a significantly higher result was obtained by the treatment of <100 nm NPs. For 250 mg/L, the tendency was the opposite, while for 150 mg/L, the results were comparable for both sizes of ZnO NPs. The results obtained by the Maskotka cultivar treated with a suspension, for which the concentration was higher than 50 mg/L, were higher for the usage of <100 nm NPs. The VI obtained by all Maskotka seeds was increased compared to the control. Those results clearly show that selecting the optimal usage of NPs is a complex process, while chosen factors have a different influence on each cultivar. It may not be possible to ensure specific conditions which would be optimal for all cultivars and the results of the vigor index illustrated it very well. For two cultivars (Maskotka and Malinowy Bossman), the most beneficial effect and the highest value were provided by the usage of <50 nm NPs at a concentration of 50 mg/L, while for the Granit cultivar, the highest result was obtained after the treatment of the 250 mg/L suspension (<50 nm). To compare, in the study of Khanm, Vaishnavi, and Shankar (2018) [20], seeds treated with 400 ppm ZnO NPs obtained a 919.8 VI, while the VI values obtained by the control was 249.97. The authors were examining the use of ZnO NPs and ZnSO_4_ in order to improve the yield and content of Zn in tomatoes. The concentrations of the used ZnO NPs suspensions were 100, 200, 400, 6000, 800, 1000, 1500, and 2000 ppm. In the case of the NPs used, only the use of the 2000 ppm ZnO NPs resulted in a decrease of the VI. These findings do not completely agree with the outcomes of our study. Although, in these two studies, different concentrations of NPs suspensions were used and different types of tomato cultivars, while our study clearly indicates the importance of the chosen type of cultivar on the final results.

## 4. Materials and Methods

### 4.1. Preparation of ZnO NPs Solutions

In this study, ZnO NPs in two sizes were used: <100 nm and <50 nm (Sigma Aldrich, Saint Louis, MO, USA). Suspension of nano ZnO were prepared with varying concentrations (50, 150, 250 mg/L) in deionized water and dispersed by ultrasonic vibration (100 W, 40 kHz) for 30 min to avoid aggregation (Bandelin Sonorex DT 102 H, BANDELIN Electronic GmbH & Co. KG, Berlin, Germany). In Table 1, the characteristic of zinc oxide NPs is demonstrated according to the reports of Sigma Aldrich company.

### 4.2. Seed Treatment Protocol

Tomato seeds of three different cultivars (*Solanum lycopersicum* L. cultivar Maskotka, cultivar Granit, cultivar Malinowy Bossman). Prior to experimentation, to avoid surface contamination, seeds were surface sterilized for 30 s by 70% ethanol, following 30 min treatment of bleach (containing 4% sodium hypochlorite), and then rinsed three times with deionized water. Next, seeds were placed in sterile Petri dishes (15 seeds on each plate) on filter paper moistened with about 2 mL of deionized water (control) or treatment solution (50, 150, 250 mg/L). The dishes were arranged in a simple randomized design with single factor and three replicates. Petri dishes were wrapped with an aluminum foil, incubated at 25 ± 2 °C, and kept in the dark for 5 days.

### 4.3. Determination of Growth Characteristics

The number of seeds germinated was counted every day after day 2. After five days of germination, the potential of seed germination was assessed in terms of seeds germination percentage (GP), mean germination time (MGT), germination index (GI), coefficient of velocity of germination (CVG), dry weight of seedlings, and vigor index (VI). The number of germinated seeds was noted daily for four days. Seeds were considered as germinated when their radicle showed at least 2 mm length.

#### 4.3.1. Germination Percentage (GP%)

Germination percentage (*GP*%) was calculated with the suitable formula:

*GP* (%) = (Number of seeds germinated/Total number of seeds) × 100

#### 4.3.2. Mean Germination Time (MGT)

Mean germination time (*MGT*) was calculated according to the following formula from the work of Matthews and Khajeh-Hosseini (2007) [29].

$$MGT\left(d\right)=\frac{\sum N\times t}{\sum N}\;$$

where *N* is the number of seeds germinated at the time of *t* and *d* is the number of days from sowing.

#### 4.3.3. Germination Rate Index (GRI)

Germination rate index (*GRI*) was calculated according to the formula given by Esechie 1994 [30]:

GRI(%/day)=∑Nt/Dt

where *Nt* is percent germination on each day (*t*) and *Dt* represents total number of days for germination. The GRI parameter reflects the percentage of germination on each day of the germination period.

#### 4.3.4. Coefficient of Velocity of Germination (CVG)

Coefficient of velocity of germination (*CVG*), which gives an indication of the rapidity of germination, was calculated in accordance with formula suggested by Jones and Sanders (1987) [31]:

$$CVG\;\left(\%/day\right)=\frac{\sum Nt}{\sum Nt\times t}/100$$

where *Nt* is number of seeds germinating on each day (*t*) and *t* represents number of days for sowing.

#### 4.3.5. Dry Weight (DW)

The study was finished after five days of seed incubation. Dry weight of seeds was determined after two days of storing them in oven at 60 °C.

#### 4.3.6. Vigor Index (VI)

Vigor index (*VI*) was calculated by the following formula by Vashisth and Nagarajan (2010) [32]. To calculate the seedling length, the representative seedlings were chosen from each exanimated group. To obtain the seedling length the ImageJ program was used.

*VI* = Germination percentage × mean of seedling length (root + shoot)

At the end of the study and when weighing the fresh weight of seedlings, plates with seedlings were placed in an oven run at 60 °C for 48 h. Then, the dry weight of seedlings was determined. Each Petri dish was treated as one replicate and all the treatments were repeated three times.

### 4.4. Data Analysis

The data were expressed as means ± standard deviation and analyzed statistically with the use of R Studio. The statistical significance of the treatments was evaluated by three factor analysis of variances (ANOVA), performed on the analyzed parameters such as germination percentage (GP), mean germination time (MGT), germination rate index (GRI), coefficient of velocity of germination (CVG), seedling vigor index (VI), and fresh and dry seedling weight. Analysis of variance was performed to determine the significance of differences between the pairs of means in different variants of the conducted experiment. The differences were statistically significant when *p* value was less than 0.05, according to Tukey statistical test, where different letters assigned to means designate a statistical difference at *p* ≤ 0.05. The chosen data were investigated through non-parametric statistics on the basis of results obtained by descriptive analysis; the means were compared by the Kruskal–Wallis test and this test highlighted significant differences.

## 5. Conclusions

In this study, we proved that factors such as ZnO NPs size and the suspension concentration are crucial for the seeds’ germination and sprouting. We further proved that the abovementioned factors affect the sprouting, but often in a different way, which depends on the examined cultivar. There are numerous studies carried out to examine the influence of ZnO NPs on different plants, including tomatoes. Still, it is not possible to indicate the optimal use of those NPs to provide the best yield and increase of important growth parameters. Most authors in their research are using ZnO NPs at different sizes, concentrations, and different application methods, thus bringing forward data which are difficult to compare. In our research, we demonstrated that another crucial feature which should be paid attention to is the type of cultivar of the chosen plant. The obtained results do not allow a choice of the optimal conditions required for all tomato cultivars to grow, although the gathered results indicate that <50 nm NPs are more beneficial for the seeds when compared to the <100 nm NPs. This conclusion is supported by the analysis of parameters such as the MGT, GRI, and CVG. In all mentioned parameters, all three cultivars showed more positive values after the treatment of <50 nm NPs, regardless of the used concentrations. This finding is very promising while the mechanism of NPs absorption is still under investigation.

In future research, we are going to extend our research by measuring the chosen parameters of growing tomato plants.

## Figures and Tables

**Table 1:** Characteristics of ZnO NPs used for the experiments.

Particle	Size [nm]	Purity [%]	Surface Area [m^2^/g]
ZnO < 50 nm	<50 nm	97.0	>10.8
ZnO < 100 nm	<100 nm	No information	10–25

## Data Availability

Not applicable.

## Sample Availability

Samples of the compounds are not available from the authors.

## Abbreviations

NPs	nanoparticles
nano-ZnO	zinc oxide nanoparticles
GP	germination percentage
MGT	mean germination time
GRI	germination rate index
CVG	coefficient of velocity of germination
VI	vigor index

## Appendix A

**Table A1:** The results of mean germination time (MGT) parameter obtained after treatment and statistic calculations. For this growth parameter, three variables were taken under consideration (NPs suspension, cultivar type, and the size of used NPs). In statistical part of the table, the statistical significance is expressed as “-” which means that *p*-value was higher than 0.05 and certain parameters had no significant influence on the results; “*” means that *p*-value is lower than 0.05, and “***” means that *p*-value is lower than 0.001. Small letters indicate the significant differences for *p*-value ≤0.05.

Cultivar	Concentration of Nps Solution	Particle Size		Control
		<50 nm	<100 nm	
Maskotka	H_2_O	-	-	3.7 ^abc^
	50 mg/L	3.2 ^abc^	4.0 ^a^	-
	150 mg/L	3.0 ^abc^	4.1 ^a^	-
	250 mg/L	3.4 ^abc^	3.8 ^abc^	-
Granit	H_2_O	-	-	3.7 ^abc^
	50 mg/L	4.3 ^ab^	3.6 ^abc^	-
	150 mg/L	3.4 ^abc^	3.9 ^a^	-
	250 mg/L	3.6 ^abc^	3.7 ^abc^	-
Malinowy Bossman	H_2_O	-	-	3.0 ^bc^
	50 mg/L	2.9 ^bc^	3.3 ^abc^	-
	150 mg/L	2.8 ^c^	3.3 ^abc^	-
	250 mg/L	2.7 ^c^	3.4 ^abc^	-
**Statistics—comparison of three variables**				
**Parameter**		***p*-value**		**Statistical significance**
NPs Concentration		0.5070		-
Cultivar type		9.87 × 10^−7^		***
Particles size		1.05 × 10^−5^		***
NPs Concentration × Particles size		0.2409		-
NPs Concentration × Cultivar type		0.5322		-
Cultivar type × Particles size		0.0419		*
NPs Concentration × Cultivar type × Particles size		0.4424		-

**Table A2:** The results of germination rate index (GRI) parameter obtained after treatment and statistic calculations. For this growth parameter, three variables were taken under consideration (NPs suspension, cultivar type, and the size of used NPs). In statistical part of the table, the statistical significance is expressed as “-”, which means that *p*-value was higher than 0.05 and certain parameters had no significant influence on the results; “*” means that *p*-value is lower than 0.05, “**” means that *p*-value is lower than 0.01, and “***” means that *p*-value is lower than 0.001. Small letters indicate the significant differences for *p*-value ≤ 0.05.

Cultivar	Concentration of NPs Solution	Particle Size		Control
		<50 nm	<100 nm	
Maskotka	H_2_O	-	-	51.58 ^fgh^
	50 mg/L	57.0 ^efgh^	50.17 ^fgh^	-
	150 mg/L	75.17 ^abcd^	52.17 ^fgh^	-
	250 mg/L	62.33 ^defgh^	55.33 ^efgh^	-
Granit	H_2_O	-	-	66.5 ^bcde^
	50 mg/L	46.75 ^h^	59.25 ^defgh^	-
	150 mg/L	61.33 ^defgh^	60.75 ^efgh^	-
	250 mg/L	52.67 ^efgh^	46.92 ^gh^	-
Malinowy Bossman	H_2_O	-	-	77.17 ^abc^
	50 mg/L	80.83 ^a^	63.75 ^cdef^	-
	150 mg/L	80.83 ^ab^	63.75 ^cdefg^	-
	250 mg/L	83.50 ^a^	65.42 ^bcdef^	-
**Statistics—comparison of three variables**				
**Parameter**		***p*-value**		**Statistical significance**
NPs Concentration		0.00413		**
Cultivar type		<2 × 10^−16^		***
Particles size		2.39 × 10^−8^		***
NPs Concentration × Particles size		0.01411		*
NPs Concentration × Cultivar type		1.32 × 10^−5^		***
Cultivar type × Particles size		8.68 × 10^−6^		***
NPs Concentration × Cultivar type × Particles size		0.04464		*

**Table A3:** The results of germination rate index (GRI) parameter obtained after treatment of <50 nm NPs and statistic calculations. In this table, two variables were taken under consideration (NPs suspension and cultivar type). In statistical part of the table, the statistical significance is expressed as “-”, which means that *p*-value was higher than 0.05 and certain parameters had no significant influence on the results; “**” means that *p*-value is lower than 0.01, and “***” means that *p*-value is lower than 0.001. Small letters indicate the significant differences for *p*-value ≤ 0.05.

Cultivar	Concentration of NPs Solution		Particle Size	Control
			<50 nm	
Maskotka	H_2_O 50 mg/L 150 mg/L 250 mg/L		- 57.0 ^cde^ 75.17 ^ab^ 62.33 ^bcd^	51.58 ^de^ - - -
Granit	H_2_O 50 mg/L 150 mg/L 250 mg/L		- 46.75 ^e^ 61.33 ^bcde^ 52.67 ^de^	66.5 ^bc^ - - -
Malinowy Bossman	H_2_O 50 mg/L 150 mg/L 250 mg/L		- 80.83 ^a^ 80.83 ^a^ 83.50 ^a^	77.17 ^a^ - - -
**Statistics—comparison of two variables**				
**Parameter**		***p*-value**		**Statistical significance**
NPs Concentration Cultivar type NPs Concentration × Cultivar type		0.00117 2.80 × 10^−13^ 2.75 × 10^−6^		** *** ***
**Cultivar**	**Concentration of NPs Solution**		**Particle Size**	**Control**
			**<100 nm**	
Maskotka	H_2_O 50 mg/L 150 mg/L 250 mg/L		- 50.17 ^cd^ 52.17 ^cd^ 55.33 ^bcd^	51.58 ^de^ - - -
Granit	H_2_O 50 mg/L 150 mg/L 250 mg/L		- 59.25 ^bcd^ 60.75 ^bcd^ 46.92 ^gh^	66.5 ^bc^ - - -
Malinowy Bossman	H_2_O 50 mg/L 150 mg/L 250 mg/L		- 63.75 ^cdef^ 63.75 ^cdefg^ 65.42 ^bcdef^	77.17 ^a^ - - -
**Statistics—comparison of two variables**				
**Parameter**		***p*-value**		**Statistical significance**
NPs Concentration Cultivar type NPs Concentration × Cultivar type		0.000766 8.51 × 10^−9^ 0.006173		*** *** **

**Table A4:** The results of coefficient of velocity of germination (CVG) parameter obtained after treatment and statistic calculations. For this growth parameter, three variables were taken under consideration (NPs suspension, cultivar type, and the size of used NPs). In statistical part of the table, the statistical significance is expressed as “-”, which means that *p*-value was higher than 0.05 and certain parameters had no significant influence on the results; “*” means that *p*-value is lower than 0.05, and “***” means that *p*-value is lower than 0.001. Small letters indicate the significant differences for *p*-value ≤ 0.05.

Cultivar	Concentration of NPs Solution	Particle Size		Control
		<50 nm	<100 nm	
Maskotka	H_2_O	-	-	28.6 ^defg^
	50 mg/L	31.2 ^abcde^	25.2 ^efg^	-
	150 mg/L	32.8 ^fg^	24.3 ^abcd^	-
	250 mg/L	29.6 ^bcdef^	26.7 ^defg^	-
Granit	H_2_O	-	-	28.6 ^defg^
	50 mg/L	22.9 ^g^	27.5 ^defg^	-
	150 mg/L	29.2 ^cdefg^	25.4 ^efg^	-
	250 mg/L	29.1 ^cdefg^	28.6 ^defg^	-
Malinowy Bossman	H_2_O	-	-	33.4 ^abc^
	50 mg/L	34.9 ^abc^	30.6 ^abcdef^	-
	150 mg/L	35.9 ^ab^	30.1 ^abcdef^	-
	250 mg/L	36.6 ^a^	29.2 ^cdefg^	-
**Statistics—comparison of three variables**				
**Parameter**		***p*-value**		**Statistical significance**
NPs Concentration		0.1913		-
Cultivar type		13 × 10^−12^		***
Particles size		2.82 × 10^−8^		***
NPs Concentration × Particles size		0.0215		*
NPs Concentration × Cultivar type		0.4077		-
Cultivar type × Particles size		9.53 × 10^−5^		***
NPs Concentration × Cultivar type × Particles size		0.0546		-

**Table A5:** The results of dry weight (DW) parameter obtained after treatment and statistic calculations. For this growth parameter, three variables were taken under consideration (NPs suspension, cultivar type, and the size of used NPs). In statistical part of the table, the statistical significance is expressed as “-”, which means that *p*-value was higher than 0.05 and certain parameters had no significant influence on the results; “***” means that *p*-value is lower than 0.001. Small letters indicate the significant differences for *p*-value ≤ 0.05.

Cultivar	Concentration of NPs Solution	Particle Size		Control
		<50 nm	<100 nm	
Maskotka	H_2_O	-	-	27.0 ^e^
	50 mg/L	26.0 ^e^	27.0 ^de^	-
	150 mg/L	28.0 ^cde^	27.0 ^e^	-
	250 mg/L	27.0 ^e^	30.0 ^cde^	-
Granit	H_2_O	-	-	45.0 ^a^
	50 mg/L	42.0 ^ab^	42.0 ^ab^	-
	150 mg/L	42.0 ^ab^	45.0 ^a^	-
	250 mg/L	42.0 ^ab^	41.0 ^ab^	-
Malinowy Bossman	H_2_O	-	-	32.0 ^cd^
	50 mg/L	36.0 ^cde^	31.0 ^cde^	-
	150 mg/L	37.0 ^abc^	33.0 ^cde^	-
	250 mg/L	29.0 ^cde^	35.0 ^bcd^	-
**Statistics—comparison of three variables**				
**Parameter**		***p*-value**		**Statistical significance**
NPs Concentration		0.1616		-
Cultivar type		<2 × 10^−16^		***
Particles size		0.3773		-
NPs Concentration × Particles size		0.0822		-
NPs Concentration × Cultivar type		0.5823		-
Cultivar type × Particles size		0.9929		-
NPs Concentration × Cultivar type × Particles size		0.0763		-

**Table A6:** The results of vigor index (VI) parameter obtained after treatment and statistic calculations. For this growth parameter, three variables were taken under consideration (NPs suspension, cultivar type, and the size of used NPs). In statistical part of the table, the statistical significance is expressed as “-”, which means that *p*-value was higher than 0.05 and certain parameters had no significant influence on the results; “*” means that *p*-value is lower than 0.05, and “***” means that *p*-value is lower than 0.001. Small letters indicate the significant differences for *p*-value ≤ 0.05.

Cultivar	Concentration of NPs Solution	Particle Size		Control
		<50 nm	<100 nm	
Maskotka	H_2_O	-	-	145.4 ^gh^
	50 mg/L	356.9 ^a^	205.9 ^de^	-
	150 mg/L	169.3 ^efg^	219.3 ^cd^	-
	250 mg/L	148.8 ^gh^	217.4 ^cd^	-
Granit	H_2_O	-	-	291.9 ^b^
	50 mg/L	113.8 ^hi^	256.3 ^b^	-
	150 mg/L	200.0 ^def^	228.2 ^cd^	-
	250 mg/L	251.4 ^bc^	93.0 ^i^	-
Malinowy Bossman	H_2_O	-	-	353.1 ^a^
	50 mg/L	324.0 ^a^	163.5 ^fg^	-
	150 mg/L	248.0 ^bc^	91.6 ^i^	-
	250 mg/L	169.8 ^efg^	117.6 ^hi^	-
**Statistics—Comparison of three variables**				
**Parameter**		***p*-value**		**Statistical significance**
NPs Concentration		<2 × 10^−16^		***
Cultivar type		1.26 × 10^−8^		***
Particles size		3.04 × 10^−16^		***
NPs Concentration × Particles size		0.0144		*
NPs Concentration × Cultivar type		<2 × 10^−16^		***
Cultivar type × Particles size		<2 × 10^−16^		***
NPs Concentration × Cultivar type × Particles size		<2 × 10^−16^		***
